# Female reproductive capacity preservation: antioxidant strategies in combating ovarian aging and cryopreservation challenges

**DOI:** 10.3389/fendo.2025.1711016

**Published:** 2026-02-27

**Authors:** Bing Xie, Kaiqi Zhang, Jiao Lin, Haiying Cheng, Xianghong Huang

**Affiliations:** 1 Reproductive Center, Xiangtan Central Hospital, Affiliated Hospital of Hunan University, Xiangtan, Hunan, China; 2 Xiangtan Central Hospital, University of South China, Hengyang, Hunan, China

**Keywords:** antioxidant interventions, female fertility preservation, ovarian aging, oxidative stress, ROS

## Abstract

The preservation of female fertility represents a pivotal area of focus within reproductive medicine, particularly in addressing ovarian damage resulting from oncological treatments and the decline in fertility associated with aging. Since the inaugural successful cryopreservation of a human oocyte in 1986, technological advancements have provided women with a form of “fertility insurance.” Nevertheless, oxidative stress exerts a profound influence on oocyte quality and the outcomes of cryopreservation. An overproduction of ROS leads to mitochondrial dysfunction, DNA damage, and chromosomal aneuploidy, especially in women of advanced reproductive age, thereby diminishing oocyte quality. Oxidative stress interferes with spindle assembly, chromosome cohesion, and spindle assembly checkpoints, thereby elevating aneuploidy rates. During the cryo-preservation process, oxidative stress prompts apoptosis and follicular loss in both oocytes and ovarian tissue, thereby undermining the success of fertility preservation efforts. Anti-oxidants such as coenzyme Q10, melatonin, and nicotinamide have been shown to mitigate oxidative stress, enhance mitochondrial function, reduce apoptosis, and im-prove the quality of oocytes and the success rates of ovarian tissue cryopreservation. This review critically examines the effects of oxidative stress on oocyte development and cryopreservation, and investigates the potential of antioxidant interventions.

## 1 Introduction

With the advancement of medical technology and the evolution of societal norms, female fertility preservation has emerged as a central concern in contemporary reproductive medicine. Since the successful birth of the first human infant from a cryopreserved oocyte in 1986, oocyte cryopreservation technology has achieved significant breakthroughs, thereby expanding the possibilities for clinical fertility preservation (1). Presently, the pressing demand in this domain primarily arises from two principal challenges: the irreversible impairment of ovarian function due to cancer therapies and the natural age-related decline in fertility. In the context of cancer treatment, while interventions such as chemotherapy and radiotherapy have markedly enhanced patient survival rates, their gonadotoxic effects can lead to ovarian failure and infertility, particularly affecting women of reproductive age and pre-pubertal girls (2). Statistics indicate that approximately 10% of newly diagnosed cancer patients globally are under the age of 45 each year, and the accelerated depletion of ovarian reserves caused by chemotherapeutic agents, such as alkylating drugs, underscores the necessity of integrating fertility preservation into treatment protocols (3). Economic and social advancements have contributed to a general postponement in the age at which women bear children. This delay is accompanied by a notable decline in oocyte quality after the age of 35, which increases the risks of miscarriage, chromosomal abnormalities, and pregnancy complications (4). Cryopreservation technology offers a form of “fertility insurance” for women in this demographic, enabling them to preserve high-quality oocytes during their reproductive prime and circumvent the biological disadvantages associated with advanced maternal age.

Oxidative stress plays a crucial role not only during the *in vitro* preservation stage but also in the reproductive decline associated with advanced maternal age (5). Within the framework of mature *in vitro* fertilization technology, the influence of advanced maternal age on fertilization and preimplantation embryo development is relatively minor; however, it significantly elevates the incidence of aneuploidy in blastocysts (6). The prevalence of aneuploidy in human oocytes increases with advancing age, rising from 2% in women in their 20s to over 50% in women over 40 years (7), with a marked escalation occurring after the age of 35. Oxidative stress is considered a primary factor contributing to chromosomal abnormalities in oocytes. The focus will be on the effects of oxidative stress on oocyte function and the preservation of oocytes and ovarian tissues.

## 2 The impact of oxidative stress on oocyte development

**Figure 1:**
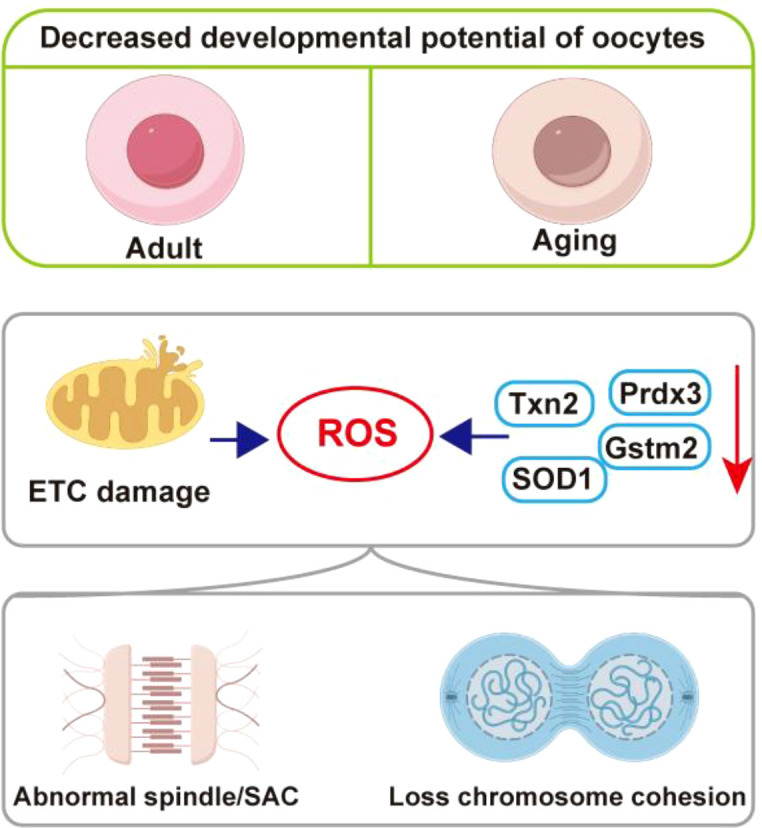
The molecular mechanism by oxidative stress leads to abnormality of oocytes. ETC, electron transport chain; ROS, reactive oxygen species; Txn2, thioredoxin 2; Prdx3, peroxiredoxin 3; Gstm2, glutathione S-transferase 2; SOD1, superoxide dismutase 1;SAC, spindle assembly checkpoint.

While advancements in assisted reproductive technology have addressed infertility issues in some younger women, they cannot fully compensate for the fertility decline observed in women of advanced reproductive age, particularly those over 40 years of age (8). A key indicator of reduced fertility in women of advanced reproductive age is the increased incidence of oocyte aneuploidy with advancing age. Oocyte aneuploidy is characterized by an abnormal number of chromosomes in oocytes, resulting from errors in chromosome segregation (6) (**Figure 1**).

**Figure 2:**
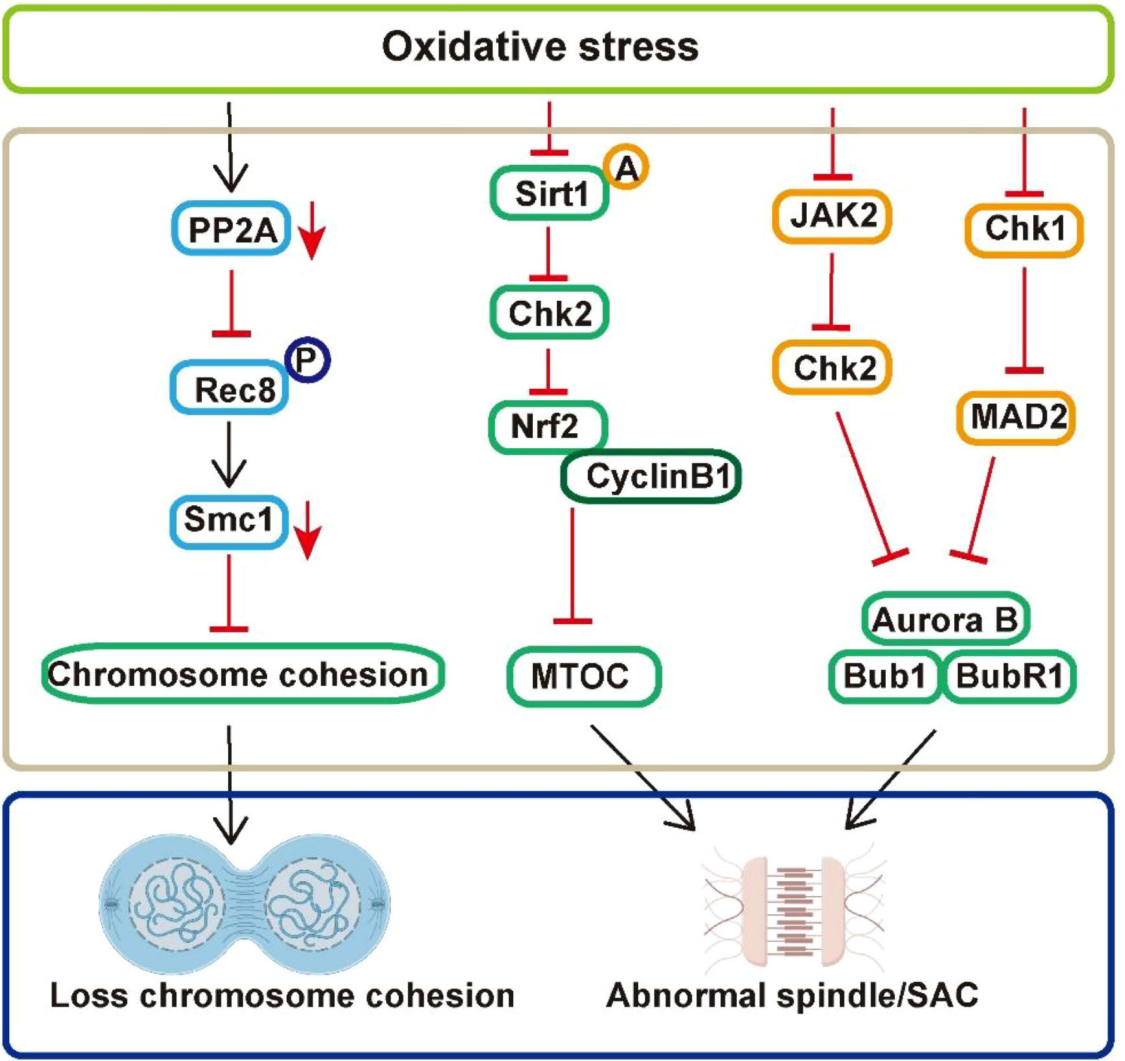
Signal pathways of oxidative stress-induced DNA damage in oocytes. PP2A, protein phosphatase 2A; MTOC, microtubule-organizing center; Mps1, monopolar spindle kinase 1; Nrf2, nuclear factor erythroid 2-related factor 2; Chk2, checkpoint kinase 2; Chk1 checkpoint kinase 1; SAC, spindle assembly checkpoint; MAD2, mitotic arrest deficient 2; APC/C, anaphase-promoting complex/cyclosome.

Oxidative stress triggers DNA damage in oocytes by compromising multiple quality control mechanisms (**Figure 2**). Initially, it disrupts chromosomal cohesion by downregulating PP2A and impairing Rec8 dephosphorylation. This destabilizes the cohesion complex (e.g., Smc1-Smc3-Mcd1-Scc1) and reduces Smc1 expression, leading to premature chromosome separation and segregation errors. In terms of spindle assembly, oxidative stress damages the microtubule-organizing center (MTOC) by altering proteins like CCP110 and TACC3. It also inhibits the Sirt1-Nrf2-Cyclin B1 axis and reduces Sirt1-dependent Chk2 acetylation, which disrupts spindle pole formation and causes chromosomal misalignment. Furthermore, oxidative stress impairs the spindle assembly checkpoint (SAC). It activates Chk1, which downregulates MAD2 and prevents its kinetochore localization, thereby allowing the APC/C to trigger premature anaphase. Concurrently, reduced levels of Aurora B, Bub1, and BubR1, along with compromised JAK2-Chk2 signaling, weaken SAC responsiveness and result in extensive aneuploidy.

### 2.1 The primary factor contributing to the deterioration in oocyte quality is the elevation in oxidative stress

In women of advanced reproductive age, oocytes demonstrate elevated levels of reactive ROS as a consequence of mitochondrial dysfunction. Oxidative stress is a critical factor contributing to the age-associated decline in oocyte quality (1). Under physiological conditions, mitochondria generate a minimal amount of ROS via the electron transport chain (ETC). The mitochondrial genome, which encodes the core components of the ETC, is particularly susceptible to ROS-induced damage due to its lack of histone protection, limited antioxidant defenses, and inefficient DNA repair mechanisms (9). Consequently, the mutation rate of mitochondrial DNA in the oocytes of women of advanced reproductive age increases, compromising the integrity of the mitochondrial ETC and precipitating oxidative stress within the oocytes (10). The age-related decline in cellular respiration further exacerbates mitochondrial electron leakage and ROS production, thereby intensifying intracellular oxidative stress. Moreover, ovarian aging significantly contributes to the accumulation of ROS within oocytes (11). The accumulation of age-related advanced glycation end products facilitates collagen cross-linking within the ovary, disrupts angiogenesis around the follicles, induces hypoxia, and promotes the accumulation of ROS within the oocytes (12). Furthermore, the antioxidant capacity of the aging ovary diminishes, accompanied by a reduction in the expression of antioxidant genes such as thioredoxin 2 (Txn2), peroxiredoxin 3 (Prdx3), superoxide dismutase 1 (SOD1), and glutathione S-transferase 2 (Gstm2), culminating in oxidative stress-induced damage within the oocytes (13). Single-cell RNA sequencing analysis reveals that, in comparison to 5-week-old mice, the expression of antioxidant genes such as human epidermal growth factor receptor 3 (Erbb3), regulator of neurocalcin 1 (Rcan1), glutathione Stransferase omega 2 (Gsto2), and methionine sulfoxide reductase B1 (Msrb1) is markedly downregulated in 32-week-old mice (14). Consequently, the mitochondrial membrane potential of oocytes decreases, ROS content significantly increases, the incidence of abnormal spindle formation rises, and the developmental potential of oocytes is reduced (15).

### 2.2 Oxidative stress results in a reduction in chromosomal cohesion

Chromosomal cohesion is facilitated by adherin proteins, which are established during the early stages of embryonic development (16). This cohesion is crucial for the accurate positioning and microtubule attachment of homologous chromosomes during the first meiotic division, thereby preventing premature separation and segregation errors (17). The proteins responsible for chromosomal adhesion form a circular complex, comprising Smc1, Smc3, Mcd1, Scc1, and Rec8, which induces chromosome condensation (18). Disruptions in this circular structure can result in the loss of chromosomal cohesion. One mechanism contributing to diminished cohesion in oocytes is age-related oxidative stress. In immature oocytes, the nucleus contains recombined homologous chromosomes that are held together by the adhesion protein complex (16). Each homologous chromosome consists of two sister chromatids joined at the centromere. During the meiotic stages MI and MII, the adhesion proteins at the centromere degrade, facilitating the separation of homologous chromosomes and sister chromatids. The adhesion protein complex deteriorates with advancing age, leading to reduced cohesion and abnormalities in chromosomal separation within oocytes (19). Rec8, a specific subunit of the adherin proteins involved in meiosis, contains a recognition site for separase, allowing it to be selectively cleaved by this enzyme. The expression of Rec8 is essential for maintaining the connection between sister chromatids until chromosome segregation occurs. Shimoi et al. (20) conducted a study on mice, revealing that oxidative stress damage in aged oocytes results in diminished expression of protein phosphatase 2A (PP2A) at the centromere, reduced dephosphorylation of the Rec8 protein, compromised protection mechanisms of adhesion proteins within the oocyte, inhibition of oocyte division and maturation, and the emergence of aneuploidy. In Drosophila oocytes with the SOD2 gene knocked out, a reduction in Smc1 expression, loss of chromosome cohesion, and a significant increase in the rate of chromosome non-separation were observed (21). These findings suggest that adhesion protein dysfunction contributes to errors in meiotic division within oocytes, corroborating that age-related oxidative stress damage in oocytes elevates the incidence of aneuploidy (21).

### 2.3 Oxidative stress results in aberrant spindle assembly

The spindle assembly in human oocytes exhibits instability. In mitotic cells, the centrosome serves as the primary microtubule-organizing center (MTOC) during spindle formation, comprising two centrioles and the surrounding pericentriolar matrix (PCM). The centrosome undergoes replication during interphase and migrates to opposite poles of the cell during mitosis, thereby initiating spindle assembly and dynamically regulating the microtubule architecture of the spindle. In human oocytes, the MTOC is constituted by four proteins located at the kinetochore and spindle microtubules: centriole coiledcoil protein 110 (CCP110), cytoskeleton-associated protein 5 (CKAP5), DISC1, and transforming acidic coiledcoil 3 (TACC3). This MTOC facilitates microtubule nucleation and spindle assembly. However, due to the absence of centrioles, the resultant spindle morphology is reduced in size, and the characteristic astral microtubules at the poles are absent, contributing to the increased instability of spindle assembly in human oocytes. Disruption of the MTOC results in defects in spindle assembly and subsequent arrest in oocyte development (22). Monopolar spindle kinase 1 (Mps1), located at the MTOC and spindle poles, plays a crucial role in MTOC aggregation and serves as the primary microtubule nucleation factor during the spindle assembly process in mouse oocytes. It accumulates at the spindle poles to ensure the formation of a stable bipolar spindle. Animal studies have indicated that the expression of Mps1 is reduced in the oocytes of older mares (over 16 years old) (23). This reduction in Mps1 expression can disrupt spindle assembly during the oocyte cleavage period in mice, leading to early embryonic DNA damage and oxidative stress (24). During the metaphase of oocyte meiosis, the nuclear transcription factor erythroid 2-related factor 2 (Nrf2) localizes to the spindle region, playing a crucial role in spindle assembly in mouse oocytes via the silencing information regulator 1 (Sirt1)-Nrf2-cell cycle protein B1 (Cyclin B1) signaling pathway. This process is particularly relevant in the context of diabetic patients, where clinical studies have demonstrated that elevated mitochondrial ROS levels can inactivate Nrf2 (24), thereby disrupting spindle assembly. Checkpoint kinase 2 (Chk2), a cell cycle checkpoint protein, is situated at the spindle poles and contributes to spindle assembly (23) During the MI and MII stages of mouse oocyte meiosis and early embryonic development, when chromosomes are properly aligned to form a spindle, Chk2 is localized at the spindle poles to facilitate spindle-related processes. However, oxidative stress has been shown to diminish the acetylation activity of Sirt1 on Chk2, thereby inhibiting Chk2 activity (25) which ultimately results in spindle assembly failure.

### 2.4 Oxidative stress leads to dysfunction of the spindle assembly checkpoint

The spindle assembly checkpoint (SAC) functions as a pivotal regulatory mechanism within the cell cycle (6). Located at the kinetochore, the SAC monitors the attachment of chromosomes to the spindle apparatus and modulates the transition from metaphase to anaphase during cell division. It accomplishes this by inhibiting the anaphase-promoting complex/cyclosome (APC/C) (25). In cases where chromosomes are not properly aligned, the SAC induces a cell cycle arrest at metaphase to prevent premature separation of chromatids. Once all chromosomes are correctly aligned and attached, the inhibition by the SAC is relieved, facilitating the equitable distribution of chromatids to daughter cells (26). The proper functioning of the SAC is crucial for the normal progression of meiosis in oocytes, and its dysfunction is a major contributor to aneuploidy in these cells. The accumulation of ROS reduces the expression of SAC-related proteins, compromises their structural integrity, destabilizes SAC function, leads to the loss of core SAC components, and promotes aneuploidy in oocytes (27). Mitotic Arrest Deficient 2 (MAD2), a crucial checkpoint signaling molecule, is situated at the kinetochore, where it plays an essential role in overseeing the attachment between spindle fibers and the kinetochore. Oxidative damage triggers the DNA damage response, which subsequently leads to a decrease in MAD2 expression and its improper localization through Chk1, resulting in aneuploidy in murine oocytes. Age-related oxidative stress is associated with reduced expression of Aurora B kinase. Since the activation of the SAC depends on Aurora B, a deficiency in Aurora B hinders the recruitment of SAC proteins to the kinetochore, weakens SAC responsiveness, and increases the occurrence of aneuploidy in murine oocytes (27). Chk2 plays a critical role in spindle assembly and the maintenance of SAC integrity during meiosis in murine oocytes. Oxidative stress reduces Chk2 activity and disrupts the JAK2-Chk2 signaling pathway, thereby impairing SAC function and leading to aneuploidy in oocytes (25). BUB1 Mitotic Checkpoint Serine/Threonine Kinase and BUB1 Mitotic Checkpoint Serine/Threonine Kinase B are critical components of the SAC signaling pathway, playing a pivotal role in SAC signal transduction. The research conducted by Riris et al. (28) revealed a significant reduction in the concentrations of Bub1 and BubR1 in the oocytes of women over the age of 37, as compared to those in women under the age of 32. This decrease negatively impacts spindle morphology and compromises the functionality of the SAC pathway, ultimately leading to erroneous chromosome segregation in oocytes (28).

### 2.5 Strategies to mitigate oxidative stress for the prevention of oocyte deterioration

As women age, reproductive senescence is linked to increased oxidative stress within the ovaries. This accumulation of reactive oxygen species (ROS) disrupts meiotic spindle assembly, impairs spindle assembly checkpoint signaling, compromises chromatin cohesion, and ultimately deteriorates oocyte quality and developmental potential in women of advanced reproductive age (17). Addressing oxidative stress is a promising therapeutic strategy to improve the quality of aging oocytes and reduce the incidence of aneuploidy (17, 18). Empirical evidence suggests that coenzyme Q10 supplementation in women over 38 years of age can reduce oxidative stress in oocytes, enhance oocyte maturation rates, and decrease aneuploidy rates following meiosis (29). Additionally, the antioxidant melatonin has been demonstrated to increase ATP levels in the oocytes of older mice (12 months old), reduce cellular apoptosis and DNA damage, prevent spindle and chromosomal abnormalities, and improve intercellular communication between oocytes and ovarian somatic cells. These effects synergistically alleviate oxidative stress–induced damage and contribute to improved oocyte competence (30). Moreover, supplementation with nicotinamide has been shown to enhance ovulation in aging mice (16 months old), restore mitochondrial function, decrease levels of ROS, maintain normal spindle and chromosomal structures, and preserve oocyte integrity (31). Recent research suggests that the administration of near-infrared cell protector-61 to mice in the late reproductive stage (10 months old) can modulate mitochondrial function, resulting in reduced ROS accumulation, preservation of spindle and chromosome structures, improved oocyte maturation, decreased aneuploidy rates, and enhanced embryo development (32). Li et al. (33)] found that intermittent fasting in older mice (14 months old) can enhance the activity of the nicotinamide adenine dinucleotide (NAD+)/Sirt1 antioxidant system, which reduces ROS accumulation, prevents DNA damage and cell apoptosis, increases follicle numbers, and improves oocyte meiotic and embryo development capabilities, thereby significantly delaying reproductive aging.

**Figure 3:**
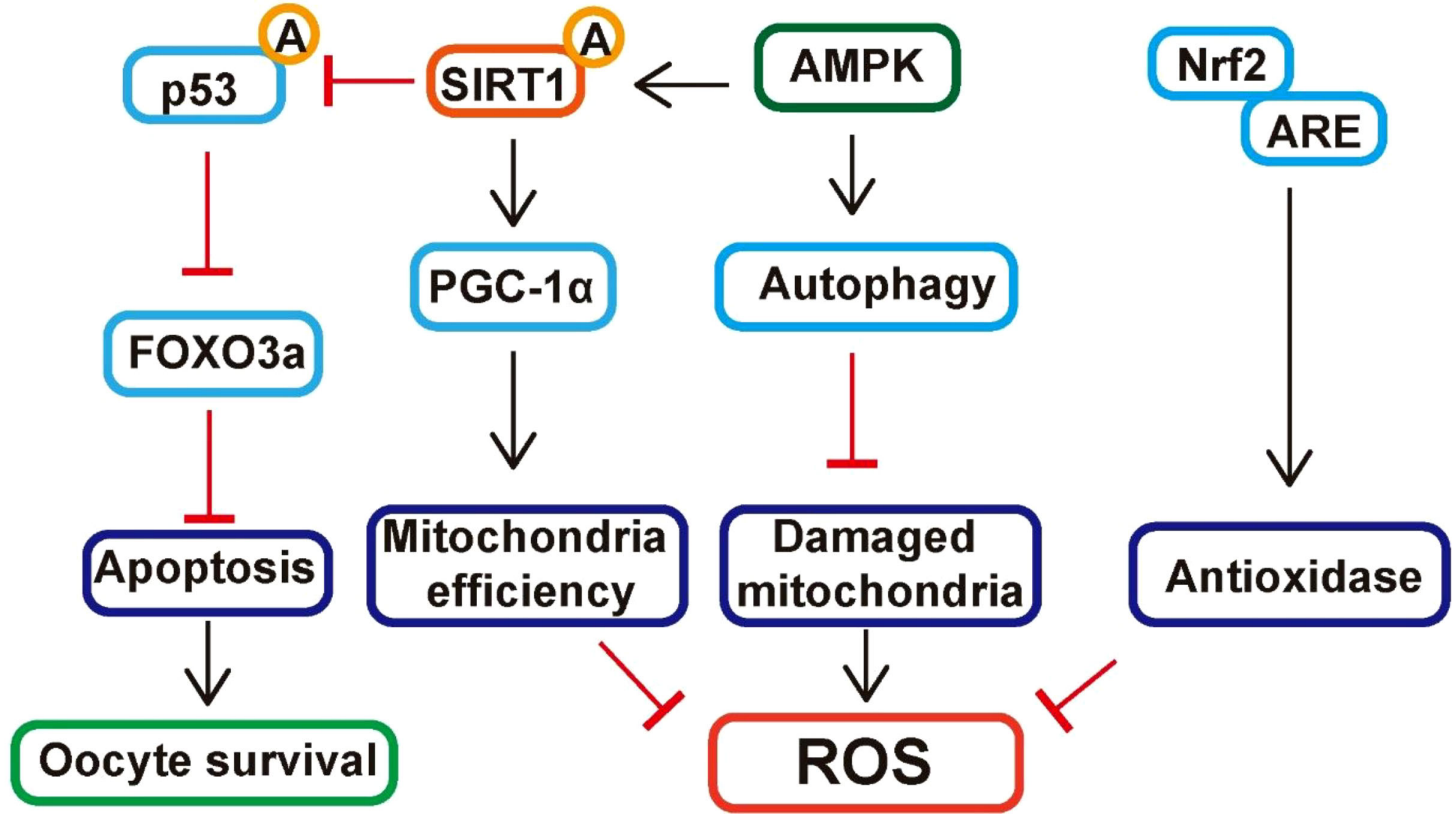
Signaling pathways that mitigate oxidative damage in oocytes.

At the molecular level, several antioxidant interventions exert their protective effects through the activation of key cellular defense pathways as shown in **Figure 3**. Activation of the Nrf2 pathway enhances the transcription of antioxidant response element (ARE)-dependent genes such as heme oxygenase-1 (HO-1), NAD(P)H quinone dehydrogenase 1 (NQO1), and glutamate–cysteine ligase (GCL), thereby promoting detoxification of ROS and maintenance of redox homeostasis in oocytes (34). Similarly, SIRT1, a NAD+-dependent deacetylase, reduces oxidative stress by deacetylating and activating peroxisome proliferator-activated receptor gamma coactivator 1-alpha (PGC-1α), which enhances mitochondrial biogenesis and antioxidant enzyme expression (35). SIRT1 also deacetylates p53 and FOXO3a, thereby inhibiting apoptosis and improving oocyte survival (36, 37). Moreover, activation of the AMP-activated protein kinase (AMPK) pathway contributes to redox balance by promoting autophagy-mediated clearance of damaged mitochondria and by upregulating Nrf2 signaling (38). AMPK–SIRT1–PGC-1α axis activation has been shown to enhance mitochondrial efficiency, reduce ROS generation, and maintain energy homeostasis within aging oocytes (39, 40). Collectively, these molecular pathways converge to attenuate oxidative damage, suppress apoptotic signaling, and preserve oocyte developmental competence during aging.

## 3 The impact of oxidative stress on the preservation of oocytes and ovarian tissues

The most effective method for the cryopreservation of oocytes and ovaries, vitrification, has garnered significant attention due to its rapid cooling capabilities (41). Vitrification facilitates the swift freezing of cells, thereby preventing damage to the cellular lipid membrane and cytoskeletal structure typically caused by ice crystal formation (41). Additionally, this technique is advantageous due to its time efficiency and cost-effectiveness, rendering it a promising tech-nology with extensive potential applications (42). Nonetheless, a primary challenge associated with vitrification cryopreservation is the oxidative stress induced by excessive ROS during the freezing and thawing processes, which adversely im-pacts cell function and viability (43).

### 3.1 The impact of oxidative stress on the cryopreservation of oocytes

Oxidative stress-induced mitochondrial dysfunction results in oocyte apoptosis. The vitrification process leads to mitochondrial dysfunction, characterized by a reduction in mitochondrial membrane potential (MMP), an increase in ROS, and elevated mitochondrial Ca^2+^ levels (44), ultimately resulting in oocyte apoptosis and diminished developmental potential (45). MMP is crucial for maintaining mitochondrial function, as it influences ATP production and normal cellular metabolism. The process of vitrification, or glassy freezing, has been shown to reduce MMP, thereby diminishing the efficiency of oxidative phosphorylation and adversely affecting mitochondrial function and oocyte development (46). Empirical evidence indicates that vitrified mouse oocytes at the germinal vesicle (GV) and metaphase II (MII) stages exhibit a significant reduction in MMP, leading to mitochondrial dysfunction (47). Alterations in MMP have a direct impact on ROS production, which can induce apoptosis and compromise the developmental potential of oocytes (48). For instance, in MII-stage mouse oocytes subjected to vitrification, both a decrease in MMP and an increase in ROS levels contribute to mitochondrial dysfunction (49). Similarly, in GV-stage mouse oocytes exposed to vitrification, there is an accumulation of mitochondrial ROS and an elevated rate of apoptosis, both of which are closely associated with mitochondrial dysfunction (47). Subsequent investigations have provided concrete evidence linking elevated mitochondrial Ca2+ levels in vitrified oocytes to impaired mitochondrial function. Specifically, studies on vitrified mouse GV-stage oocytes revealed a significant accumulation of mitochondrial Ca2+ and ROS, which was accompanied by a marked decrease in MMP and ATP levels, ultimately leading to mitochondrial dysfunction (50, 51). These ionic and metabolic imbalances are critical because they trigger downstream apoptotic pathways. It has been observed that such mitochondrial impairments facilitate the opening of the mitochondrial permeability transition pore (mPTP), causing the release of cytochrome C (Cyt C) from the intermembrane space (52). Consistently, Qin et al. (53) detected significantly increased Cyt C levels in vitrified mouse MII-stage oocytes, which correlated with early apoptosis. Similarly, in a bovine model, vitrified MII-stage oocytes exhibited elevated expression of the apoptotic protein Cyt C compared to fresh controls, contributing to reduced survival rates (54).

Mechanistically, oxidative stress compromises mitochondrial integrity primarily through the activation of the ASK1–JNK–Bcl-2/Bax apoptotic axis. Specifically, Excess ROS oxidize thioredoxin (Trx), causing dissociation and activation of apoptosis signal-regulating kinase 1 (ASK1). Active ASK1 phosphorylates downstream MKK4/7 which in turn activates JNK. Activated JNK translocates to mitochondria and phosphorylates anti-apoptotic Bcl-2 family members (e.g., Bcl-2 phosphorylation at serine residues such as Ser70) leading to functional inactivation of Bcl-2/Bcl-xL, and promotes pro-apoptotic conformational changes and mitochondrial translocation of Bax (55, 56). These phosphorylation events (PTMs) destabilize the mitochondrial outer membrane, promote mitochondrial outer membrane permeabilization (MOMP), cytochrome c release and caspase activation, and directly reduce mitochondrial membrane potential (Δψm) and ATP generation (57). High ROS levels induce reversible oxidative PTMs on critical thiol residues of ETC proteins (for example S-glutathionylation or S-nitrosylation of Complex I subunits), impairing electron transport efficiency and increasing electron leakage that further amplifies ROS production and lowers Δψm (58). Concomitantly, ROS can oxidatively activate Ca^2+^/calmodulin-dependent protein kinase II (CaMKII) via methionine oxidation (e.g., Met281/282 oxidation), producing sustained kinase activity that phosphorylates mitochondrial Ca^2+^ handling proteins such as the mitochondrial calcium uniporter (MCU) or its regulatory subunits. Phosphorylation of MCU enhances mitochondrial Ca^2+^ uptake, precipitating mitochondrial Ca^2+^ overload, opening of the mitochondrial permeability transition pore (mPTP), loss of Δψm, release of pro-apoptotic factors and progressive mitochondrial dysfunction (59).

### 3.2 The impact of oxidative stress on the cryopreservation of ovarian tissue

The cryopreservation and transplantation of ovarian tissue are not constrained by the patient’s age or timing. For prepubescent females or patients with malignant tumors who cannot postpone radiotherapy or chemotherapy, cryopreservation and transplantation of ovarian tissue represent the sole method for fertility preservation (60). Cryopreservation involves the surgical removal of ovarian tissue via laparoscopy or laparotomy, excluding the medulla while retaining the cortex, which is then sectioned into slices approximately 1 mm thick and 5–10 mm in length and width. These slices are preserved in liquid nitrogen through vitrification or controlled-rate freezing (slow freezing). This technique was first applied to humans in 1996 (61). In 2004, Donnez et al. (62) documented the birth of the first child following *in situ* transplantation of cryopreserved ovarian tissue. Since then, the incidence of pregnancies and live births among patients undergoing ovarian tissue cryopreservation and transplantation has increased significantly (63), with over 200 infants born through this technique (64). Since 2019, the American Society for Reproductive Medicine has classified ovarian tissue cryopreservation as an established, non-experimental method for fertility preservation (65). Ovarian tissue transplantation addresses fertility issues and partially restores endocrine function in patients (66). Nevertheless, this procedure encounters significant challenges, such as the potential contamination of ovarian tissue with malignant cells, which heightens the risk of tumor recurrence (10). Additionally, cryopreservation and transplantation processes can result in damage, leading to a reduction in ovarian reserve and a shortened lifespan of the transplanted tissue (67). The longevity of the transplanted ovarian tissue is contingent upon the survival of primordial follicles (68), with 50% to 90% of these follicles being lost post-transplantation. Consequently, research into the mechanisms of ovarian damage following transplantation and the development of protective strategies is of paramount importance.

It is well documented that the processes of cryopreservation and transplantation induce significant oxidative stress in ovarian tissue, a phenomenon closely paralleling the impact of ROS observed in cryopreserved oocytes (69, 70). This oxidative stress can adversely affect oocyte fertilization rates, embryo quality, and clinical pregnancy outcomes by inducing follicular apoptosis and causing aberrant ultrastructural morphology (69). The detrimental effects of low-temperature preservation of ovarian tissue encompass ROS generation, DNA fragmentation, and caspase activation, which may result in damage to granulosa and stromal cells, oocyte degeneration, and follicular attrition. Excessive ROS production not only impairs cellular function but also activates primordial follicles, potentially leading to depletion of the ovarian follicle reserve through the inhibition of PTEN and subsequent activation of Akt pathways (70). Notably, the oxidative damage induced by ischemia-reperfusion injury following ovarian tissue transplantation is particularly pronounced. The initial hypoxic insult and subsequent ischemia-reperfusion injury during transplantation are critical contributors to follicular depletion (71). In the absence of vascular anastomosis during ovarian tissue transplantation (72), the blood and oxygen supply primarily relies on the neovascularization within the transplanted ovarian tissue. Prior to the establishment of new vasculature, the transplanted tissue is limited to acquiring a portion of its necessary oxygen and nutrients through diffusion from adjacent tissues (73). Consequently, the tissue is susceptible to ischemia and hypoxia (74). Hypoxic conditions in the transplanted ovarian tissue can disrupt mitochondrial membrane potential homeostasis and induce endoplasmic reticulum stress, thereby initiating an apoptotic cascade that leads to increased apoptosis of follicles and stromal cells.

**Figure 4:**
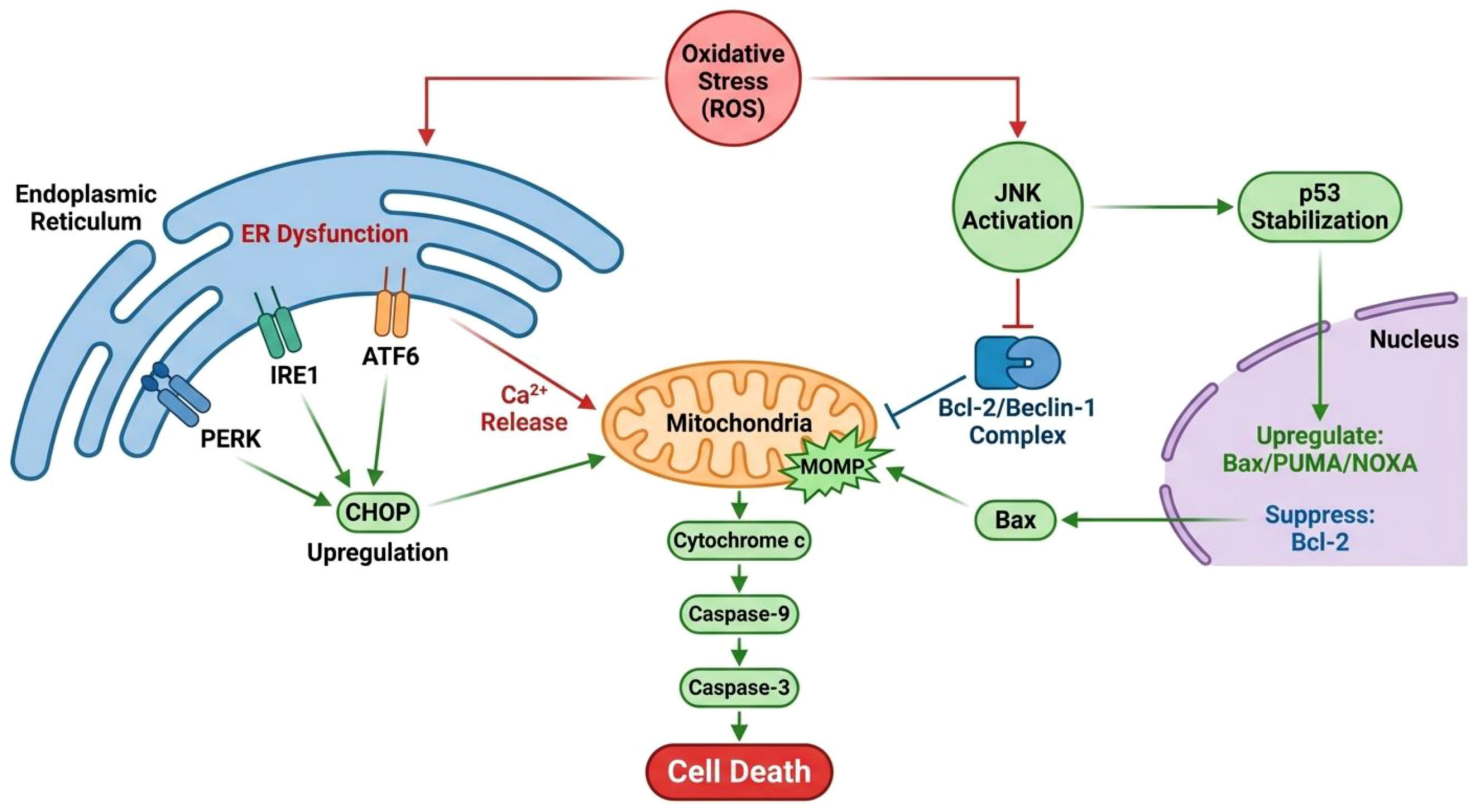
Crosstalk between endoplasmic reticulum (ER) stress and mitochondrial dysfunction in oxidative stress-induced cell death.

Mechanistically, oxidative stress triggers endoplasmic reticulum stress through the activation of the PERK-eIF2α-ATF4 and IRE1α-XBP1 signaling pathways, as well as through the upregulation of CHOP (C/EBP homologous protein), which promotes apoptotic signaling (75) as shown in **Figure 4**. ROS can also disturb protein folding within the ER lumen by altering the redox balance of disulfide bond formation, further activating the unfolded protein response (UPR) via PERK, IRE1, and ATF6 sensors (76). Persistent activation of these pathways leads to calcium leakage from the ER, mitochondrial dysfunction, and caspase-dependent apoptosis. Moreover, oxidative stress can activate the ROS–JNK–p53 signaling pathway, which plays a central role in promoting apoptosis under oxidative and endoplasmic reticulum stress conditions. Excessive ROS activates c-Jun N-terminal kinase (JNK), which subsequently phosphorylates and stabilizes the tumor suppressor p53. Activated p53 translocate to the nucleus, where it upregulates pro-apoptotic genes such as Bax, PUMA, and NOXA, while suppressing anti-apoptotic Bcl-2 expression (77). The increased Bax/Bcl-2 ratio promotes mitochondrial outer membrane permeabilization and cytochrome c release, leading to the activation of caspase-9 and downstream executioner caspase-3 (78). Simultaneously, JNK-mediated phosphorylation of Bcl-2 disrupts its association with Beclin-1, enhancing both apoptosis and autophagic cell death (79). These processes collectively amplify the apoptotic signaling cascade triggered by endoplasmic reticulum stress, resulting in extensive follicular and stromal cell loss following transplantation.

Concurrently, the ATP deficiency resulting from hypoxia promotes the disintegration of organelles and cell membranes, producing substantial amounts of oxygen free radicals and inflammatory mediators, which further intensify cellular damage and apoptosis (80). Granulosa and stromal cells exhibit higher levels of proliferation and metabolism compared to primordial oocytes, rendering them more vulnerable to the effects of transplantation and subsequent apoptosis. This suggests that a significant portion of follicular loss in transplanted ovarian tissue may be attributed to the extensive apoptosis of granulosa and stromal cells (66). The revascularization process of transplanted ovarian tissue necessitates a specific duration; functional blood vessels form within 7 days in mouse models and 10 days in human tissue (74), at which point oxygen pressure stabilizes (81). Upon restoration of the blood supply to the transplanted ovarian tissue, a substantial production of reactive oxygen species occurs, leading to vascular endothelial dysfunction, increased microvascular permeability, tissue edema, and an intensified inflammatory response. These factors collectively contribute to further cellular and tissue damage, as well as apoptosis (74).

### 3.3 Antioxidant interventions for the preservation of ovarian tissue and oocyte in the context of *in vitro* fertilization

Oxidative stress triggers apoptosis in granulosa cells, leading to the upregulation of pro-apoptotic factors and the downregulation of anti-apoptotic proteins, ultimately causing ovarian tissue damage and follicular depletion (34). To counteract oxidative damage during cryopreservation or reperfusion, antioxidants provide protective effects via multiple mechanisms, including the scavenging of excessive ROS, activation of endogenous antioxidant pathways, inhibition of mitochondrial dysfunction, and modulation of apoptosis-related gene expression as shown in **Table 1** (39). Strategies for application involve incorporating antioxidants into the freezing/thawing medium to directly protect the grafts or enhancing antioxidant capacity through systemic interventions in the host (43). These approaches collectively contribute to the reduction of inflammatory mediators, a decrease in lipid peroxidation products, and improvements in follicle survival and transplantation success rates. Resveratrol primarily acts via the SIRT1–PGC-1α axis, enhancing mitochondrial biogenesis, increasing ATP synthesis, and reducing ROS generation. It also deacetylates FOXO3a and p53, thereby suppressing apoptosis and improving oocyte survival (82, 83). Melatonin, on the other hand, activates both the Nrf2–ARE and AMPK–SIRT1 pathways, leading to upregulation of antioxidant enzymes such as HO-1, NQO1, and SOD, stabilization of mitochondrial membrane potential, and inhibition of cytochrome c release (84, 85). Quercetin attenuates oxidative damage by stimulating Nrf2 translocation to the nucleus and concurrently inhibiting NF-κB–mediated inflammatory signaling, which together mitigate ROS-induced apoptosis (86, 87). Coenzyme Q10 enhances mitochondrial electron transport efficiency and indirectly activates AMPK signaling, thus maintaining energy homeostasis and preventing mitochondrial depolarization (88). In addition, glutathione (GSH) and N-acetylcysteine (NAC) restore intracellular redox equilibrium by replenishing GSH pools and activating AMPK–Nrf2–HO-1 signaling, which promotes ROS detoxification and suppresses apoptotic cascades (89). Sericin exerts its protective effects by upregulating Nrf2 expression and enhancing endogenous antioxidant enzyme activity, thereby preserving cellular membrane integrity and preventing caspase-dependent apoptosis (90). Collectively, these agents converge on the Nrf2, SIRT1, and AMPK signaling networks to restore redox homeostasis, prevent mitochondrial dysfunction, and inhibit oxidative stress–induced apoptosis, ultimately improving follicular viability and oocyte developmental competence during cryopreservation and transplantation.

**Table 1:** Antioxidant interventions for the preservation of ovarian tissue and oocyte.

Tissue	Components	Types	Effects	References
Oocyte	1 μmol/L Resveratrol	Porcine germinal vesicle stage oocytes	Significantly increased the mitochondrial membrane potential	(83)
	2 μmol/L Resveratrol	Porcine metaphase II oocytes	Inhibit cell apoptosis	(94)
	10-9mol/L Melatonin	Bovine oocyte	Reduce intracellular ROS levels and inhibit cell apoptosis.	(85)
	10–7 mol/L Melatonin	Mouse germinal vesicle stage oocytes	Maintain mitochondrial function and increase the survival rate of oocytes	(53)
	5 μmol/L Quercetin	Sheep germinal vesicle stage oocytes	Reduce DNA damage after freezing and thawing, alleviate cell apoptosis	(87)
	50 μmol/L Coenzyme Q10	Cow germinal vesicle stage oocyte mother cell	Relieve the apoptosis of oocytes after freezing	(88)
Ovarian	0.1 mmol/L Melatonin	Rat ovarian tissue	Reduce oxidative stress and cell apoptosis in ovarian tissues	(95)
	0.1 mmol/L Melatonin	Rat ovarian tissue	Reduce the levels of oxidative damage markers in ovarian tissue	(96)
	100 mg/kg glutathione and 1250 U/kg ulinastatin	Human ovarian tissue	The levels of IL-6, TNF-αand MDA decreased.	(97)
	1% Sericin	Mouse ovarian tissue	Inhibit cell apoptosis, reduce MDA, and enhance endogenous antioxidant enzymes Level	(90)
	75 mmol/L N-acetylcysteine	Human ovarian tissue	Increase the levels of antioxidant enzymes and alleviate oxidative stress	(98)

It is noteworthy that the effectiveness of antioxidant strategies may vary depending on the type of cryoprotectant used (e.g., dimethyl sulfoxide, ethylene glycol, or glycerol) and the species investigated. In cryopreservation systems containing permeable cryoprotectants, antioxidants may exert enhanced efficacy by facilitating intracellular penetration and direct scavenging of ROS, whereas in non-permeable cryoprotectant systems, their action predominantly relies on extracellular radical neutralization (91, 92). Moreover, interspecies differences in ovarian tissue architecture, mitochondrial density, and endogenous antioxidant capacity can lead to variable outcomes, with certain antioxidants demonstrating stronger protective effects in rodent models compared to human or ruminant tissues (93). Therefore, the development of complementary antioxidant strategies tailored to the specific cryoprotectant formulation and species physiology may maximize the efficiency of ovarian tissue cryopreservation and transplantation.

## 4 Conclusion

Oxidative stress presents a significant challenge in the preservation of female fertility, profoundly affecting oocyte development, cryopreservation success rates, and ovarian tissue transplantation. Both aging and the cryopreservation process contribute to the accumulation of ROS, leading to mitochondrial dysfunction, chromosomal abnormalities, and apoptosis, which collectively diminish oocyte quality and the survival rate of ovarian tissue. Antioxidant interventions targeting oxidative stress, such as the supplementation of coenzyme Q10, melatonin, nicotinamide, and the implementation of intermittent fasting strategies, have demonstrated potential in enhancing oocyte quality, reducing aneuploidy incidence, and improving the success rates of ovarian tissue transplantation. Future research should focus on optimizing antioxidant application methods in cryopreservation and transplantation to improve the efficacy and safety of fertility preservation techniques, thereby offering more reliable fertility options for cancer patients and women of advanced reproductive age.
